# Extrication as a Predictor of Outcome in Motor Vehicle Crashes

**DOI:** 10.7759/cureus.77225

**Published:** 2025-01-10

**Authors:** Zachary M Daniels, Andrew McCague, Austin Henken-Siefken

**Affiliations:** 1 Trauma and Acute Care Surgery, Desert Regional Medical Center, Palm Springs, USA

**Keywords:** extrication, glasgow coma scale, injury severity score, intensive care unit, mechanical ventilation, motor vehicle collision, prognosis, trauma

## Abstract

Background

Motor vehicle accidents are one of the most common causes of severe injury and death worldwide. The process of assisted removal, performed by emergency medical services, is termed extrication. Extrication is only warranted in severe collisions, which leads one to assume it may be associated with worse outcomes. The goal of this study is to review existing literature regarding extrication as a marker of prognosis and investigate whether or not this trend is seen in our level 1 trauma registry.

Methodology

We conducted a retrospective cohort study on patients from our trauma registry between 1/1/2016 and 1/1/2021. During this time 3,318 patients met trauma activation criteria following a motor vehicle collision and were then categorized based on their extrication status. We queried the dataset for demographic information (gender, race, ethnicity, age) and markers of poor prognosis (admission rate, ICU admission rate, GCS, average ISS, average ventilator days) and compared rates of extrication within each of these groups. Using Microsoft Excel, we ran Student t-tests to calculate p-values for quantitative variables and chi-squared tests to calculate p-values for qualitative variables, to assess for statistical significance.

Results

Around 36.32% of extricated patients required admission to the medical floor, compared to 32.73% of non-extricated patients; 35.47% of extricated patients required admission to the ICU whereas 17.30% of non-extricated patients required ICU admission. There was a higher rate of death in the emergency department in extricated patients compared with non-extricated patients with 2.14% of extricated patients requiring transfer to the morgue, and 0.84% of non-extricated patients. The p-value for the T-Test comparing extrication among these various dispositions was < 0.005 indicating a statistically significant association between extrication and disposition. The rate of presentation with a GCS of less than 9 was higher in extricated patients; 12.44% of extricated patients presented with a GCS of less than 9 compared with 3.93% of non-extricated patients. This p-value was < 0.005 indicating a statistically significant association between extrication and GCS. Extricated patients had a higher injury severity score on average (13.21) compared to non-extricated patients (7.09), they had longer duration of mechanical ventilation (9.75 days) compared to non-extricated patients (6.21), longer average ICU stays (8.31 days) compared to non-extricated patients (4.99 days), and longer hospital admissions (11.01 days) compared to non-extricated patients (5.97 days). These variables were independently assessed via Student t-test and found to have p-values < 0.005 indicating statistically significant associations between extrication and average ISS, duration of mechanical ventilation, and length of stay in the ICU and hospital.

Conclusions

Vehicle extrication following motor vehicle collisions demonstrated a statistically significant association with markers of poor prognosis including rates of medical floor admission, ICU admission, death in the emergency department, likelihood of GCS < 9, higher injury severity score, longer duration requiring mechanical ventilation, and longer ICU and general hospital stays. Understanding that vehicle extrication is associated with poor prognosis will inform providers working at trauma centers that these patients are more likely to require critical care services.

## Introduction

Approximately 1.3 million people per year die as a result of motor vehicle collisions according to the World Health Organization [1]. Furthermore, 40% of these people will be trapped in their vehicles and require assisted removal [2]. This assisted removal is performed by emergency medical personnel and is referred to as extrication. When the injury severity is mild, or when the vehicle damage is minimal, self-extrication is typically the preferred option because it minimizes spinal motion and time spent in the vehicle. If spinal motion and entrapment time can be reduced, this is typically associated with more favorable outcomes for patients [3]. However, when self-extrication is not a realistic option, the two most common methods of assisted extrication include the Kendrick extrication device (KED) and the Rapid Extrication (RE) technique [4]. In comparison to self-extrication, extrication using either the KED or RE technique is associated with more spinal movement and thus worse prognosis [5]. The goal of this study is to summarize and analyze current research on extrication as a predictor of prognosis in motor vehicle collisions using data from a level 1 trauma center and assess whether or not there was a statistically significant association between markers of poor prognosis and rates of extrication in our sample.

## Materials and methods

We conducted a retrospective cohort study using a de-identified dataset on emergency department visits from 01/01/2016 through 01/01/2021 at Desert Regional Medical Center, a level 1 trauma center in Palm Springs, California. Approval was obtained from Metro West Medical Center Institutional Review Board (approval number: 2024 - 022). We queried our data set to identify all patients that met trauma activation criteria following a motor vehicle collision (MVC), and then categorized based on extrication status (extricated vs not extricated). We determined that there were 3693 patients seen in our emergency department who met trauma activation criteria and were involved in a motor vehicle collision. Of these 3693, the extrication status was recorded on 3318 patients. We queried the dataset for demographic information and markers of poor prognosis. The specific variables recorded include: demographic information (age, gender, sex, race, ethnicity), Injury Severity Score (ISS), length of hospital stay, whether or not they required intensive care unit (ICU) admission, days requiring mechanical ventilation, Glasgow Coma Scale (GCS), and outcome/disposition. We then calculated rates of extrication within each of these groups. For quantitative variables, we used a Student t-test in Microsoft Excel (Microsoft Corp., Armonk, NY) to calculate p-values and thus assess statistical significance. For qualitative variables, we used a chi-squared test in Microsoft Excel to calculate p-values and thus assess statistical significance. We considered p-values less than 0.05 to be statistically significant.

## Results

Our dataset from Desert Regional Medical Center, a level 1 trauma center in Palm Springs, CA documents 3,318 patients from our trauma registry that met trauma activation criteria following motor vehicle collisions (MVC) and were documented to have either required extrication or not from January 1st, 2016 - January 1st, 2021. Of these 3,318 patients involved in an MVC, 468 (14.10%, N = 3318) required extrication and 2850 (85.90%, N = 3318) did not require extrication.

When comparing rates of extrication among males and females, 246 (52.66%, N = 468) of extricated patients were male and 222 (47.44%, N = 468) were female, whereas 1542 (54.12%, N = 2850) of non-extricated-patients were male and 1306 (45.82%, N = 2850) were female. The P-value was calculated and found to be 0.53 demonstrating no statistically significant association between gender and rates of extrication (Table 1).

**Table 1:** Differences in rates of extrication among males and females. Chi square test was used to calculate p value and thus determine statistical significance.

Gender	Extricated	Not Extricated	Chi Square Value	P value
Male	246 (52.56%)	1542 (54.12%)	0.403	0.53
Female	222 (47.44%)	1306 (45.82%)		

We also compared rates of extrication among the elderly (greater than or equal to 65 years of age) and the non-elderly and found that 66 (14.10%, N = 468) of extricated patients were elderly and 402 (85.90%, N= 468) were not elderly, whereas 2291 (80.39%, N = 2850) of not extricated patients were not elderly and 559 (19.61%, N = 2850) were elderly. The p-value was calculated to be < 0.005 demonstrating a statistically significant association between age and rate of extrication (Table 2).

**Table 2:** Differences in rates of extrication among the elderly and non-elderly. Elderly (greater or equal to 65 years of age). Chi square test was used to calculate p value and thus determine statistical significance.

Age	Extricated	Not Extricated	Chi Square Value	P value
Younger than 65	402 (85.90%)	2291 (80.39%)	7.99	< 0.005
Greater or equal to 65 years old	66 (14.10%)	559 (19.61%)		

We also compared rates of extrication among those of Hispanic/Latino ethnicity and those that are not of Hispanic/Latino ethnicity. We found that 211 (45.09%, N = 468) of extricated patients were Hispanic/Latino and 251 (53.63%, N = 468) were not Hispanic/Latino. Among not extricated patients, 1305 (45.79%, N = 2850) were Hispanic/Latino and 1509 (52.95%, N = 2850) were not Hispanic/Latino. The p-value was calculated to be 0.77 demonstrating the association between ethnicity and extrication rate is not statistically significant (Table 3).

**Table 3:** Differences in rates of extrication among Hispanics/Latinos and Non-Hispanics/Latinos. Chi square test was used to calculate p value and thus determine statistical significance.

Ethnicity	Extricated	Not Extricated	Chi Square Value	P value
Hispanic/Latino	211(45.09%)	1305 (45.79%)	0.079	0.77
Non-Hispanic/Latino	251(53.63%)	1509 (52.95%)		

There was also a comparison of extrication rates among patients of various races. Among extricated patients, 259 (55.34%, N = 468) were white, 11 (2.35%, N = 468) were Asian, 28 (5.98%, N = 468), 28 (5.98%, N =468) were African American, 3 (0.64%, N = 468) were Native Hawaiian/Pacific Islander, and 161 (34.40%, N = 468) identified as “other” race. Among non-extricated patients 1672 (58.67%, N =2850) were white, 60 (2.11%, N = 2850) were Asian, 179 (6.28%, N = 2850) were African American, 15 (0.53%, N = 2850) were Native Hawaiian/Pacific Islander, and 923 (32.39%, N = 2850) identified as “other” race. The p-value was calculated and found to be 0.83, demonstrating the association between extrication rate and race is not statistically significant (Table 4).

**Table 4:** Differences in rates of extrication among different races. The chi-square test was used to calculate the p-value and thus determine statistical significance.

Race	Extricated	Not extricated	Chi-square value	P-value
White	259 (55.34%)	1672 (58.67%)	1.48	0.83
Asian	11 (2.35%)	60 (2.11%)		
African American/Black	28 (5.98%)	179 (6.28%)		
Native Hawaiian/Pacific Islander	3 (0.64%)	15 (0.53%)		
Other Race	161 (34.40%)	923 (32.39%)		

All patients were immediately seen in the emergency department and the disposition upon leaving the emergency department was recorded for all patients. Regarding the extricated patients, 55 (11.75%, N = 468) were discharged, 170 (36.32%, N = 468) were admitted to the medical floor, 166 (35.47%, N = 468) were admitted to the Intensive Care Unit (ICU), 56 (11.97%, N = 468) required surgery and were taken directly to the operating room (OR), 10 (2.14%, N = 468) were deceased in the emergency department and taken directly to the morgue, 2 (0.43%, N = 468) left against medical advice, 1 (0.21%, N = 468) was admitted to an inpatient psychiatric facility, 2 (0.43%, N = 468) were discharged to correctional facility/court/law enforcement, 0 were admitted to pediatric unit, and 6 (1.28%, N = 468) were transferred. Regarding non-extricated patients, 1123 (39.40%, N = 2850) were discharged, 933 (32.73%, N = 2850) were admitted to the medical floor, 493 (17.30%, N = 2850) were admitted to the ICU, 139 (4.88%, N = 2850) required surgery and were taken directly to the OR, 24 (0.84%, N = 2850) were deceased in the emergency department and taken directly to the morgue, 34 (1.19%, N =2850) left against medical advice, 5 (0.18%, N = 2850) were admitted to an inpatient psychiatric facility, 45 (1.58%, N = 2850) were discharged to correctional facility/court/law enforcement, 2 (0.07%, N = 2850) were admitted to the pediatric unit, and 51 (1.79%, N = 2850) were transferred. The p-value was calculated and found to be < 0.005, demonstrating that the association between post-ED disposition and extrication status is statistically significant (Table 5).

**Table 5:** Differences in post-emergency department disposition among the extricated versus non-extricated. Against medical advice (AMA). The chi-square test was used to calculate the p-value and thus determine statistical significance.

Post ED Disposition	Extricated	Not extricated	Chi-square value	P-value
Discharge	55 (11.75%)	1123 (39.40%)	202.76	< 0.005
Medical floor	170 (36.32%)	933 (32.73%)		
Intensive care unit	166 (35.47%)	493 (17.30%)		
Operating room	56 (11.97%)	139 (4.88%)		
Morgue	10 (2.14%)	24 (0.84%)		
Left AMA	2 (0.43%)	34 (11.93%)		
Inpatient psychiatric facility	1 (0.21%)	5 (0.18%)		
Correctional facility/court/law enforcement	2 (0.43%)	45 (1.58%)		
Pediatric unit	0 (0%)	2 (0.07%)		
Transfer	6 (1.28%)	51 (1.79%)		

The Glasgow Coma Scale (GCS) was calculated for 3,295 patients of our 3,318 patient sample size. As for the extricated patients, 58 (12.44%, N = 468) had a GCS of less than 9, and 408 (87.55%, N = 468) had a GCS of 9 to 15. As for the non-extricated patients 112 (3.96%, N = 2850) had a GCS of less than 9, and 2717 (96.04%, N = 2850) had a GCS of 9 to 15. The p-value was calculated to be < 0.005 demonstrating that the association between GCS and extrication status is statistically significant (Table 6).

**Table 6:** GCS upon arrival to the emergency department. Glasgow Coma Scale (GCS). The chi-square test was used to calculate the p-value and thus determine statistical significance.

GCS on arrival	Extricated	Not extricated	Chi-square value	P-value
Less than 9	58 (12.44%)	112 (3.96%)	58.9	< 0.005
9 to 15	408 (87.55%)	2717 (96.04%)		

The average Injury Severity Score (ISS) was calculated for each patient. The average ISS for extricated patients was 13.21 with a standard deviation of 9.52. The average ISS for non-extricated patients was 7.09 with a standard deviation of 7.36. The p-value was calculated and found to be < 0.005 demonstrating the association between average ISS and extrication status is statistically significant. Of the patients that were admitted to the intensive care unit, the number of days each patient required mechanical ventilation was recorded. The average number of days on the ventilator for extricated patients was 9.75 and 6.21 for non-extricated patients. The p-value was calculated to be < 0.005 demonstrating that the association between average days spent on the ventilator and extrication status is statistically significant. Of the patients that were admitted to the intensive care unit, the length of stay (LOS) was recorded. Of the extricated patients that were admitted to the ICU, they spent on average 8.31 days in the ICU versus 4.99 days for non-extricated patients that were admitted to the ICU. The p-value was calculated to be < 0.005 demonstrating that the association between ICU length of stay and extrication status is statistically significant. For patients that were admitted to the hospital following a motor vehicle collision, the length of stay was recorded. Extricated patients spent on average 11.01 days in the hospital whereas non-extricated patients spent on average 5.97 days in the hospital. The p-value was calculated to be < 0.005 demonstrating the association between hospital length of stay and extrication status is statistically significant (Table 7).

**Table 7:** Average values for ISS, days on a ventilator, ICU LOS, and general hospital LOS in extricated vs non-extricated patients. Injury Severity Score (ISS); LOS; length of stay; standard deviation (SD). The student t-test was used to calculate the p-value and thus determine statistical significance.

Extrication status	Average ISS	SD	P-value
Extricated	13.21	9.52	< 0.005
Not extricated	7.09	7.36	
Extrication Status	Average Ventilator Days	SD	P value
Extricated	9.75	11.55	< 0.005
Not Extricated	6.21	8.25	
Extrication Status	Average ICU Days	SD	P Value
Extricated	8.31	10.21	< 0.005
Not Extricated	4.99	5.99	
Extrication status	Average Hospital Days	SD	P-value
Extricated	11.01	11.85	< 0.005
Not extricated	5.97	7.28	

The average injury severity score was calculated and compared across age groups. The average ISS for extricated non-elderly patients was 13.49 compared to 7.07 for non-extricated non-elderly patients. The p-value was calculated to be < 0.005 demonstrating that the association between average ISS and extrication status in the non-elderly population is statistically significant. In contrast, the average ISS for elderly extricated patients was 11.53 and 7.16 for elderly non-extricated patients. The p-value was calculated to be < 0.005 demonstrating that the association between extrication status and average ISS in the elderly population is also statistically significant (Table 8).

**Table 8:** Average ISS among those less than 65 years of age and those 65 or older in extricated and non-extricated groups. Injury Severity Score (ISS). Student t-test was used to calculate p-value and thus determine statistical significance.

Extrication Status	Average ISS Age < 65	SD	P-value
Extricated	13.49	9.74	< 0.005
Not Extricated	7.07	7.53	
Extrication Status	Average ISS Age greater or equal to 65	SD	P-value
Extricated	11.53	7.86	< 0.005
Not Extricated	7.16	6.65	

## Discussion

It is important for healthcare providers working at trauma centers to be aware that vehicle extrication is a marker of poor prognosis. This is often one of the first pieces of information that emergency department physicians receive when they are alerted of a patient who was involved in a motor vehicle collision and are en route to the ED. If a provider understands that the extricated patient is more likely to have a low GCS, require intubation and ICU admission, or might even be deceased upon arrival, then they will be mentally prepared to provide critical care services and have the appropriate specialists notified and available to provide a timely response.

To better understand the process of extrication and why it might be associated with poor patient outcomes, we must first review the steps taken by emergency medical services (EMS) during an extrication and the specific devices they use. Patients involved in motor vehicle collisions often experience neck and back injuries, meaning that spinal immobilization is an important consideration for EMS. As previously discussed, the KED and RE are the two most common extrication techniques practiced in the field. The KED is a semi-rigid device that is fastened to a patient’s torso, maintains the seated position in which they were found, and thus limits spinal movement. RE on the other hand is a method of removing a patient from a vehicle that involves rapidly moving them through a series of coordinated motions from sitting to a supine position. This technique is generally used in unstable patients prior to immobilization via a rigid backboard. To apply a KED requires moving the patient which can cause further pain and potential exacerbation of the possible spinal injury. Compared to RE, it also is a much longer process which places the crew at increased exposure to potentially hazardous conditions on scene, and delays transport time to the hospital [4].

Extrication has been included in the CDC trauma activation guidelines because of its documented role in improving patient outcomes in trauma. This is because it allows EMS personnel to easily perform life-saving interventions such as airway management and blood product transfusion [6].

Furthermore, it has been routinely demonstrated that patients who are rapidly transported to a hospital following a motor vehicle collision have a higher chance of survival, especially in the context of severe injuries [1]. This highlights the importance of including RE techniques in the protocol for trauma response if they have been shown to cut down on delays in transport time. One example is the Norwegian Chain method, which was demonstrated to be capable of reliably removing entrapped victims in motor vehicle wreckage in less than 20 minutes, a significant improvement in comparison to the previous standard [7].

Of the 3318 patients from our trauma registry who were involved in motor vehicle collisions, 53.89% were male compared to 46.05% female. Of the men that were involved in MVCs, 13.76% (N = 1,788) required extrication whereas 14.52% (N = 1,528) of female patients required extrication. This is contradictory to our theory that extrication is a predictor of prognosis given that it has been routinely demonstrated that men are far more likely than women to be involved in fatal motor vehicle collisions [8]. We would have expected a higher rate of males requiring extrication compared to female patients if it were true that extrication is associated with increased injury severity and thus poor prognosis. However, as demonstrated in Table 1, the association between gender and extrication status in our sample was not found to be statistically significant. It is possible that if this study were repeated with a larger sample size we would find results consistent with the literature. However, it is also possible that our study did not demonstrate an association between extrication and age/gender because of adequate variability in rates of extrication within these two sub-groups. Furthermore, when comparing the rates of extrication among the elderly and the non-elderly, within our population there was a 10.56% (N=625) rate of extrication among the elderly compared to 14.93% (N =2693) among the non-elderly. This is also contradictory to our theory that extrication is a predictor of prognosis given the literature supporting an increased risk of fatal injury in the elderly compared to the non-elderly [9].

Attributable risk, the difference between the risk of an outcome occurring in exposed versus unexposed individuals, is used to measure the excess risk of an outcome that can be attributed to exposure in cohort studies [10]. For patients involved in motor vehicle collisions, there was a statistically significant increased risk of 3.59% in extricated patients compared to non-extricated patients. This is consistent with the theory that extrication is a predictor of poor prognosis. Patients who are admitted to the medical floor are deemed by the admitting physician too unstable to be addressed on an outpatient basis, which signifies a more severe injury with a worse prognosis. The intensive care unit is reserved for critically ill patients requiring mechanical ventilation, high doses of vasopressor medications for hemodynamic support, and the highest level of care available at any given hospital. In our population, extrication carries with it a statistically significant increased risk of ICU admission of 18.17%, indicating increased injury severity and thus a worse prognosis. It has been routinely demonstrated that car accidents frequently lead to ICU admission and are in fact among the most common causes of trauma admissions to the ICU [11]. ICU admission is generally associated with higher mortality rates compared to medical floor admission and thus demonstrates a worse prognosis compared to non-extricated patients. Furthermore, our study found that there was a statistically significant increased risk of dying in the emergency department thus requiring transfer to the morgue in extricated vs non-extricated patients with an attributable risk of 1.3%, which inherently indicates a worse prognosis in extricated patients.

The Glasgow Coma Scale is used to assess neurological function in states of altered consciousness due to various medical conditions. A score of less than 8 is classically used to delineate between severe head injury and is associated with a worse prognosis [12]. In our retrospective cohort study, we found that extricated patients had a statistically significant 8.48% increased risk of having a GCS of 9 or less. The injury severity score (ISS) is an index that is calculated using the single highest abbreviated injury score for the three most severely injured body regions in a trauma patient. It has been considered the gold standard indicator for anatomical injury severity since its introduction in 1974 and has excellent predictive value for prognostic factors such as length of hospital stay and ICU admissions [13]. Our retrospective cohort study demonstrated a statistically significant increase in average ISS when comparing extricated and non-extricated patients. There was also a higher average number of days requiring mechanical ventilation, higher average duration of ICU care, and longer length of stay when comparing extricated patients to non-extricated patients. The increased risk of GCS of less than 9, higher ISS, and increased duration in terms of mechanical ventilation, ICU care, and general hospital care associated with extricated patients all cumulatively help to further demonstrate the increased risk of poor prognosis associated with extrication.

It is imperative to address the limitations of this study. The dataset fails to specify the methods by which patients were extricated which has large implications on the degree of patient movement during extrication, and how long the process delayed transport time. There is also no mention of why a patient required extrication. It is possible that some patients were extricated simply because of significant deformity of the vehicle following the collision and thus they required emergency medical personnel assistance, or if it was due to severe injury. Additionally, the dataset does not specify if extricated patients include those who were able to self-extricate following EMS intervention (i.e. if a patient was able to self-extricate/ambulate after the fire department removed the door or roof of the vehicle). Another limitation is the small sample size, which may potentially impact the ability of the study to detect trends that would be more easily perceived with a larger sample size.

It is important for healthcare providers working at trauma centers to understand the implications when a patient presents to the emergency department following a motor vehicle collision and it is known that they were extricated from the vehicle. Once it is known that the patient has been extricated, providers should communicate directly with emergency medical response staff to understand which method was used to extricate and how this impacted the degree of movement during extrication and the time spent on-scene to adequately perform the extrication. This study adequately demonstrates a statistically significant association between markers of poor prognosis and vehicle extrication, supporting the existing literature on the topic and underscoring the importance of trauma providers knowing their patients’ extrication status.

## Conclusions

In summary, it was found that upon review of our de-identified data set of emergency department visits post-motor vehicle collision at our level 1 trauma center, patients that required extrication were associated with markers that are indicative of poor prognosis when compared to patients that did not require extrication. Our study purely demonstrates an association between extrication and poor prognosis, indicating that patients who sustain more serious injuries are more likely to require extrication and thus have worse prognosis. These markers include increased likelihood of hospital and ICU admission, increased length of stay on average, increased duration requiring mechanical ventilation, higher average injury severity score, and increased risk of having a GCS < 9 on admission. These findings are consistent with the existing literature and help to further inform providers that work at trauma centers that patients involved in motor vehicle collisions that required extrication are more likely to have severe injuries requiring intensive care.
